# Biomarkers for the diagnosis and post-Kasai portoenterostomy prognosis of biliary atresia: a systematic review and meta-analysis

**DOI:** 10.1038/s41598-021-91072-y

**Published:** 2021-06-03

**Authors:** Lin He, Dennis Kai Ming Ip, Greta Tam, Vincent Chi Hang Lui, Paul Kwong Hang Tam, Patrick Ho Yu Chung

**Affiliations:** 1 Department of Radiotherapy, Tangdu Hospital, Air Force Military Medical University, Xi’an, China; 2 School of Public Health, Li Ka Shing Faculty of Medicine, The University of Hong Kong, Pok Fu Lam, Hong Kong SAR; 3 Departmet of Surgery, Li Ka Shing Faculty of Medicine, The University of Hong Kong, Pok Fu Lam, Hong Kong SAR

**Keywords:** Biomarkers, Gastroenterology

## Abstract

To evaluate the accuracy of biomarkers for the early diagnosis of biliary atresia (BA) and prognostic stratification after Kasai portoenterostomy (KPE). We conducted a systematic review of PubMed, Web of Science, Embase, Scopus and OVID for English literature reporting BA biomarkers published before August 2020. Screening, data extraction, and quality assessment were performed in duplicate. A total of 51 eligible studies were included in the systematic review, and data from 12 (4182 subjects) were extracted for meta-analysis regarding the following 2 domains: (1) serum matrix metallopeptidase-7 (MMP-7), interleukin33 (IL-33) and γ-glutamyl transferase (GGT) to differentiate BA from non-BA; (2) the aspartate aminotransferase to platelet ratio index (APRi) to predict post-KPE liver fibrosis/cirrhosis. The summary sensitivity, specificity and area under the curve (AUC) of MMP-7 for diagnosing BA were 96%, 91% and 0.9847, respectively, and those of GGT were 80%, 79% and 0.9645, respectively. The summary sensitivity and specificity of IL-33 for diagnosing BA were 77% and 85%, respectively. The summary sensitivity and specificity of APRi for predicting post-KPE liver fibrosis were 61% and 80%, respectively, and the summary sensitivity, specificity and AUC of APRi for predicting post-KPE cirrhosis were 78%, 83% and 0.8729, respectively. Moreover, good evidence was shown in investigations of serum IL-18 and IL-33 in distinguishing BA from healthy controls, serum IL-18 for prognosis of post-KPE persistent jaundice, and serum hyaluronic acid and MMP-7 for prognosis of post-KPE significant liver fibrosis. MMP-7, IL-33 and GGT are useful biomarkers to assist in the diagnosis of BA. APRi might be used to predict post-KPE significant liver fibrosis and cirrhosis. These noninvasive biomarkers can be integrated into the management protocol of BA.

## Introduction

Neonatal jaundice is common, and up to two-thirds of all newborns develop this problem within the first two weeks of life^1^. While most cases are classified as physiological jaundice that is self-limiting without long-term sequalae, more serious disorders are not uncommon. For example, biliary atresia (BA), choledochal cyst, Alagille syndrome and progressive familial intrahepatic cholestasis are common pathological causes of neonatal cholestasis requiring prompt surgical treatment. Approximately 80% of infants with pathological jaundice in need of surgical intervention also have BA^2^, which requires the most urgent surgical attention, as there is a narrow treatment window for this condition. Therefore, distinguishing BA from other causes of neonatal cholestasis (non-BA) is essential for an optimal outcome.

The occurrence of BA varies widely among different populations, with the highest incidence rates in Asia^3^. If untreated, affected infants develop progressive liver disease and die within the first two years of life^4^. A timely and uneventful Kasai portoenterostomy (KPE) can potentially restore bile flow in 30%-80% of BA patients, but complications do occur^5^. It is estimated that up to 56%-74% of post-KPE BA patients at 10 years of age require liver transplantation^6^. Despite significant advances in the management of BA, establishing an early diagnosis for it and predicting post-KPE outcomes remain two major challenges. An early diagnosis will lead to a timely operation, which may promote surgical success rate^7^. Currently, an accurate diagnosis can only be established by invasive methods such as intraoperative cholangiography and liver biopsy^8^; diagnosis can be confirmed by examination of the liver tissue obtained at the time of KPE^9,10^. Postoperatively, however, there is a lack of objective parameters for predicting a poor prognosis. Our objective in this study is to identify noninvasive biomarkers to promote early diagnosis and identification of patients with poor prognosis based on a systematic review and meta-analysis of reported serum biomarkers.

## Materials and methods

This systematic review and meta-analysis followed the Preferred Reporting Items for Systematic Reviews and Meta-analyses guidelines^11,12^. No Ethical or Institutional Review Board approval was required for the study design.

### Literature search

We conducted a computerized search in the PubMed, Web of Science, Embase, Scopus and OVID databases to identify English-language articles relevant to our objective up to August 1^st^, 2020. The following terms were used: ((“biliary atresia”[MeSH]) OR (biliary atresia)) AND (biomarker) AND (diagnosis OR prognosis OR portoenterostomy).

### Inclusion and exclusion criteria

Studies evaluating the application of serum biomarkers for early diagnosis or post-KPE prognosis were considered eligible for our analysis. In addition, the articles met the following inclusion criteria: (1) populations—for diagnosis, both BA patients and non-BA and/or healthy control (HC) groups were compared and for prognosis, BA patients were assessed after KPE; (2) reference standard— BA diagnoses or post-KPE prognostic outcomes were compared using standard measurements, including liver function and native liver survival rate. Potential citations that met any of the following criteria were excluded: (1) article type—animal experiments, reviews, case reports and case series including less than 10 patients, editorials, letters, comments and conference papers; (2) biomarkers that required liver biopsy/intraoperative cholangiography/laparoscopy; and (3) overlapping study populations.

### Data extraction

The following data were extracted from the included studies using a standardized form: (1) study characteristics—last name of the first author, publication year, study duration, country of origin, study type, and number of patients; (2) demographic characteristics—age, percentage of males; (3) biomarker characteristics—name, test sample, test method, test timeframe and cutoff value; and (4) outcome characteristics—positive or negative correlation of biomarkers and diagnostic performance or prognostic outcomes, and the sensitivity, specificity, positive likelihood ratio (PLR), negative likelihood ratio (NLR), diagnostic odds ratio (DOR) and area under the curve (AUC) of the biomarkers with/without an identified cutoff value.

### Quality assessment

The methodological quality of the articles included for the meta-analysis was assessed using our tailored questionnaires in terms of the Quality Assessment of Diagnostic Accuracy Studies 2 (QUADAS-2) criteria^13^. Two reviewers (PHY Chung and L He) independently assessed the literature search, study selection, data extraction, and quality assessment. If there were any inconsistences, they were addressed by a third reviewer (PKH Tam).

### Data synthesis and statistical analysis

The primary outcome of our study was the performance of biomarkers for the early diagnosis of BA and/or post-KPE prognosis. The ultimate purpose of this study was to identify biomarkers that promote an early diagnosis or prediction of post-KPE prognosis. We constructed 2 × 2 contingency tables based on the extracted true and false positives and negatives from available studies. Summary estimates of diagnostic test accuracy data, including sensitivity, specificity, PLR, NLR and DOR with their 95% confidence intervals (CIs), were calculated by the Mantel–Haenszel method (fixed effect model) or the DerSimonian-Laird method (random effect model)^14^. A hierarchical summary receiver operating characteristic (SROC) curve with its 95% confidence region was plotted. Of note, several statistical methods were employed to evaluate any possible bias in our meta-analysis, as follows. (1) The threshold effect—computation of the Spearman correlation coefficient between the logit of sensitivity and logit of 1-specificity; a strong positive correlation (Spearman correlation coefficient > 0.6; *p* < 0.05) would indicate a considerable threshold effect^15,16^. (2) Heterogenicity—heterogenicity that represented the degree of variability in results across the included studies, as evaluated by Cochran’s Q test and I^2^ test^17^. The *p* value of Cochran’s Q test < 0.10 suggested significant heterogeneity and different cutoff intervals of I^2^ values at 0–25%, 25–50%, 50–75% and 75–100%, respectively corresponding to nonsignificant, moderate, substantial and considerable heterogeneity. The heterogenicity of the hierarchical SROC curve was calculated by weighted regression with the Inverse Variance method (Moses’ model), and the result of the heterogenicity test determined the pooling model selection. (3) Because of the small number of studies included, the calculation of publication bias was not possible. An AUC of SROC greater than 0.7 indicated a high predictive accuracy for the biomarker^18^. The diagnostic meta-analysis was conducted by Meta-Disc 1.4 software, version 1.4 (http://www.hrc.es/investigacion/metadisc_en.htm,).

## Results

### Literature search

**Figure 1:**
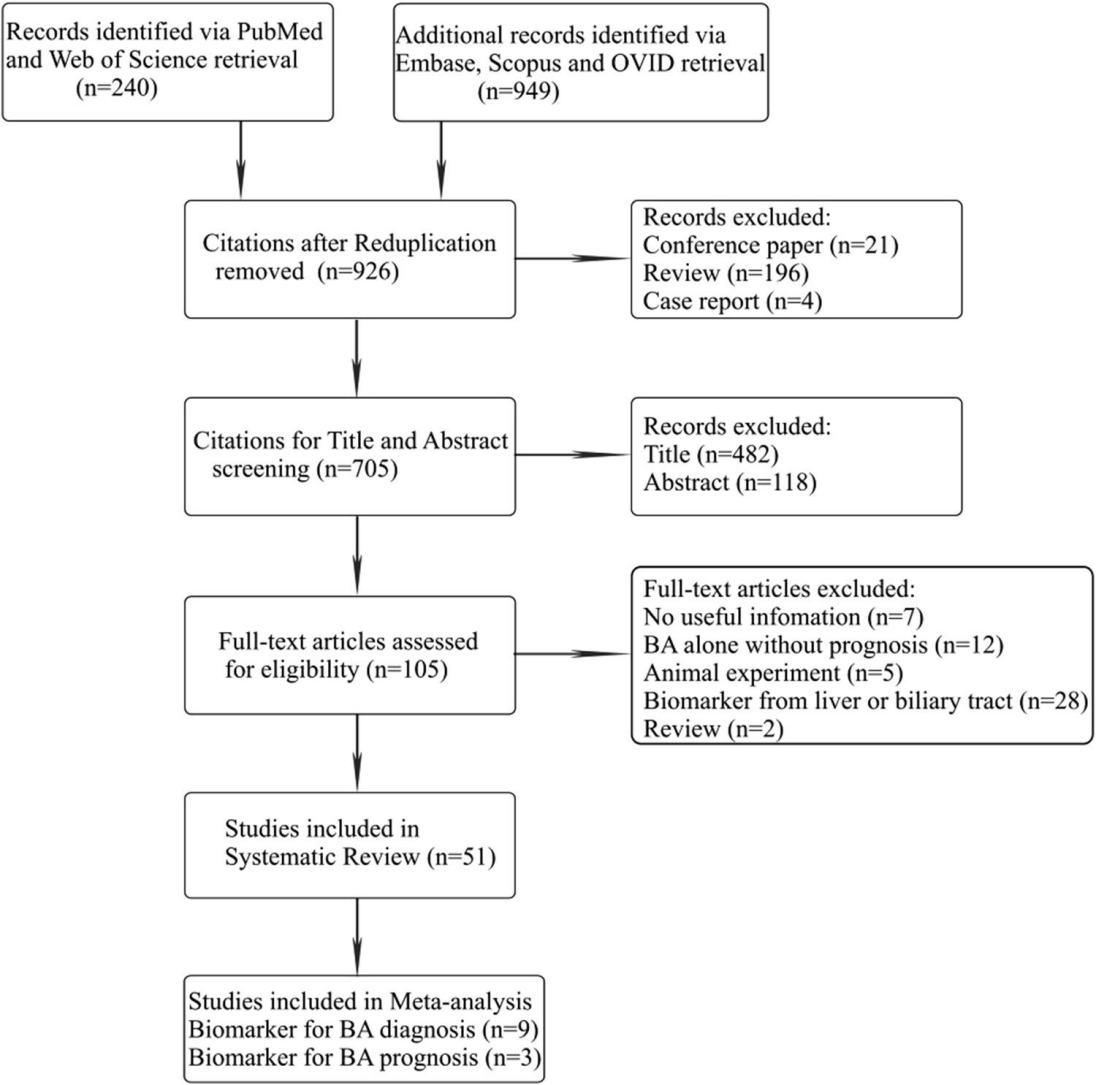
Flow diagram of study selection. Abbreviations: BA, biliary atresia.

A flow diagram of the literature screening selection is outlined in Fig. 1. Of the 1,189 citations obtained from the PubMed, Web of Science, Embase, Scopus and OIVD databases, 263 duplications, 21 conference papers, 196 reviews and 4 case reports were excluded. The remaining 705 citations were examined by title and abstract screening, and 600 of them were removed. After full-text assessment of the remaining 105 studies, 54 were further omitted for the following reasons: (1) 7 articles had no useful information; (2) 12 involved BA alone without prognosis; (3) 5 were animal experimental studies; (4) 28 used biomarkers for the liver or biliary tract; and (5) 2 were reviews. Ultimately, 51 articles were considered eligible for systematic review^19–69^. Thirty-one studies^20–23,30–55,67^ reported single-center evidence of biomarkers related to BA diagnosis or post-KPE prognosis, and 8 studies^24–29,64,65^ provided multicenter evidence but were unable to be included in the meta-analysis. The data from the remaining 12 studies were entered into 2 × 2 contingency tables, and the performance of biomarkers for early diagnosis^19,56–60,66,68,69^ or post-KPE prognosis of BA^61–63^ was evaluated. All biomarkers that are useful for the identification of BA diagnosis or post-KPE prognosis are summarized in eTable 1 (*Supplementary materials, pages 1–2*). More studies are needed to reaffirm the performance of these biomarkers because only evidence from individual case–control studies or case series was documented.

### Quality assessment of the studies included for meta-analysis

**Figure 2:**
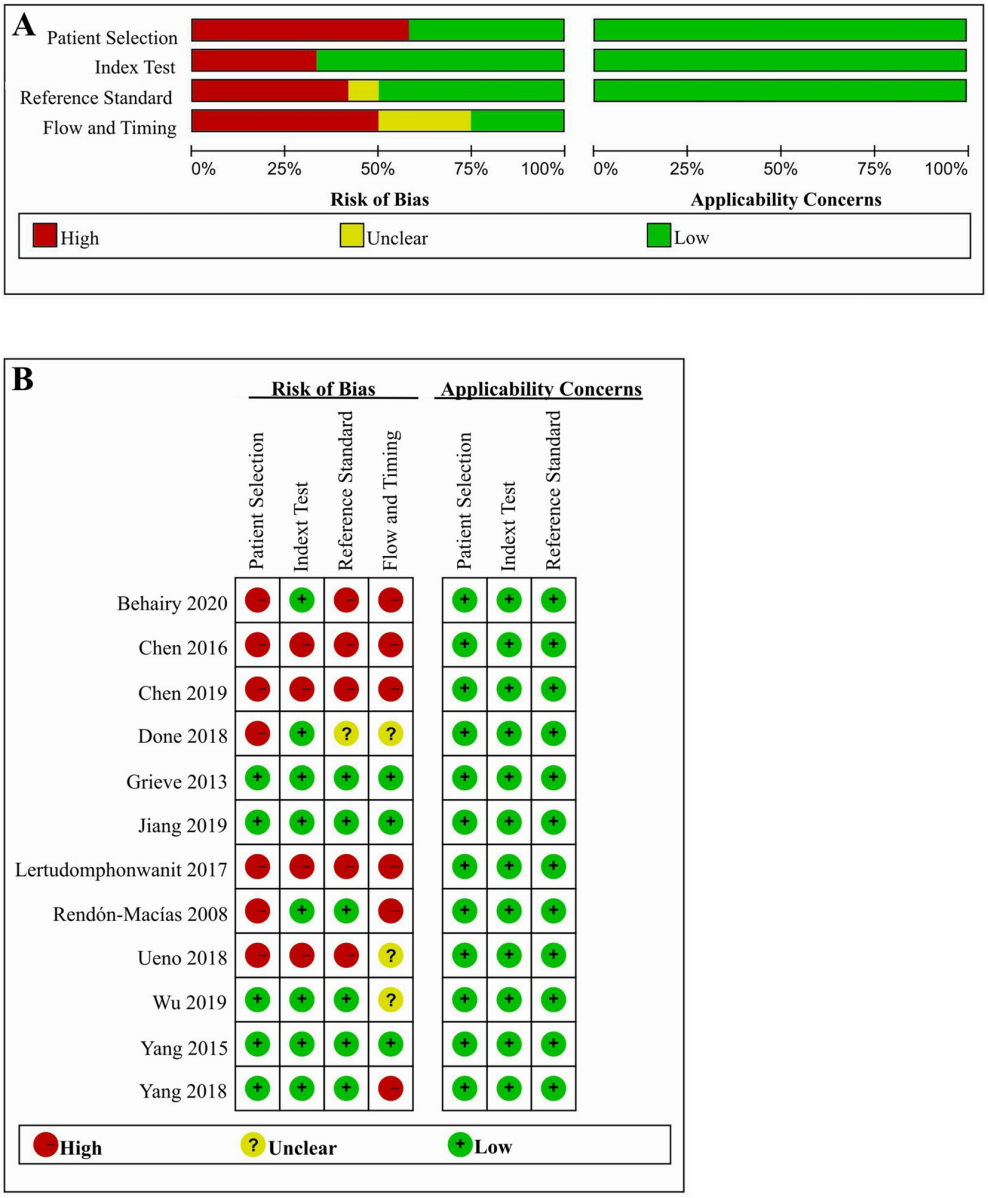
Quality assessment of the included studies for meta-analysis based on QUADAS-2 criteria. (**A**) Methodological quality graph; (**B**) Methodological quality summary. ‘ − ’ in red, ‘ + ’ in green and ‘?’ in yellow mean high risk, low risk and unclear risk, respectively.

The quality assessment of the 12 included studies based on the QUADAS-2 criteria is shown in Fig. 2. In the patient selection domain, there was a high risk of bias in 7 studies because they did not avoid a case–control design^19,56,59,63,66,68,69^. Four studies showed a high risk of bias in the index test domain because their index test results were not interpreted without knowledge of the results of the reference standard^19,59,63,69^. In addition, five studies showed a high risk of bias in the reference standard domain due to a lack of reciprocal blinding between the index test and the reference standard^19,56,59,63,69^. For the flow and timing domain, there was a high risk of bias for six articles because intervals between the index test and the reference standard were not appropriate^19,56,58,59,66,69^; the other 3 articles showed an unclear risk of bias because these intervals were not clearly reported^57,63,68^. Applicability concerns were found for none of the 12 studies.

### Characteristics of the studies included for meta-analysis

The study and biomarker characteristics of the included studies are presented in Table 1. The study characteristics in “patient-level” analysis and the demographic characteristics of the included studies are summarized in Table 2. The publication date of the 12 included articles ranged from 2008 to 2020 (median: 2018).; the number of case–control studies was identical to that of retrospective cohort studies (n = 5), and more than half of the included studies were conducted in China^19,57,58,60,62,68,69^. We retrieved 4 studies investigating the diagnostic performance of matrix metallopeptidase-7 (MMP-7)^57–60^, 2 investigating that of interleukin 33 (IL-33)^19,56^ and 3 investigating that of γ-glutamyl transferase (GGT)^59,68,69^ in the early differentiation of BA from non-BA; 3 studies evaluating that of aspartate aminotransferase to the platelet ratio index (APRi) for post-KPE prognosis (specifically, 2 for significant liver fibrosis and 3 for cirrhosis)^61–63^. Additionally, Table 1 summarizes the test method and test timeframe of biomarkers, and Table 2 represents the percentage of males and mean age of the analyzed subjects in individual biomarker analysis.

**Table 1:** Characteristics of included studies in the “study-level” analysis.

Study	Publication year	Study duration	Original nation	Biomarker	Test method	Test time	No. of Cases	No. of Controls	Ref
**Biomarkers for differentiating BA to non-BA**									
Wu	2019	2008–2018	China	MMP-7	ELISA	After KPE	36	64	^57^
Yang	2018	2016–2018	China	MMP-7	ELISA	Before KPE	75	60	^58^
Lertudomphonwanit	2017	NA	USA	MMP-7/GGT	ELISA	Before/at KPE	35	35	^59^
Jiang	2019	2017–2018	China	MMP-7	ELISA	Before KPE	187	101	^60^
Behairy	2020	2017–2019	Egypt	IL-33	ELISA	After KPE	30	30	^56^
Chen	2019	2016–2017	China	IL-33	ELISA	Before KPE	31	45	^19^
Rendón-Macías	2008	2000–2002	Mexico	GGT	NA	Before KPE	12	17	^66^
Chen	2016	2014–2016	China	GGT	NA	Before KPE	1189	119	^69^
Dong	2018	2012–2017	China	GGT	NA	Before KPE	1512	216	^68^
**Biomarkers for predicting post-KPE significant fibrosis**									
Ueno	2018	2015–2018	Japan	APRi	NA	Regardless of time	11	26	^63^
Yang	2015	2010–2013	China	APRi	NA	Before KPE	56	35	^62^
**Biomarkers for predicting post-KPE cirrhosis**									
Grieve	2013	1999–2010	UK	APRi	NA	At KPE	28	232	^61^
Yang	2015	2010–2013	China	APRi	NA	Before KPE	10	81	^62^
Ueno	2018	2015–2018	Japan	APRi	NA	Regardless of time	11	26	^63^

Abbreviations: BA, biliary atresia; MMP-7, matrix metallopeptidase-7; IL-33, interleukin 33; GGT, γ-glutamyl transferase; APRi, aspartate aminotransferase to platelet ratio index; post-KPE, after Kasai portoenterostomy; ELISA, enzyme-linked immunosorbent assay; CLEIA, chemiluminescent enzyme immunoassay.

**Table 2:** Characteristics of the included studies in the “patient-level” analysis.

Characteristic	Studies, No. (%) (N = 12)	Analyzed subjects, No. (%) (N = 4182)
**Study type**		
Prospective cohort	1 (8.33)	260 (6.22)
Case–control study	5 (41.67)	2093 (50.05)
Retrospective cohort	5 (41.67)	1792 (42.85)
Unknown cohort	1 (8.33)	37 (0.88)
Publication date, median (range), y	2018 (2008–2020)	–
**Biomarkers**		
MMP-7	3 (25.00)	523 (12.51)
MMP-7/GGT	1 (8.33)	70 (1.67)
GGT	3 (25.00)	3065 (73.29)
IL-33	2 (16.67)	136 (3.25)
APRi	3 (25.00)	388 (9.28)
Mean age of subjects in MMP-7 studies, median (range), d*	case: 58.085 (42.36–62) control: 58.935 (44.23–68.1)	–
Male sex of subjects in MMP-7 studies, median (range), %	case: 44.31 (38.89–54.29) control: 75.125 (58.3–82.86)	–
Mean age of subjects in IL-33 studies, median (range), d*	case: 97.115 (56.23–138) control: 100.92 (54.84–147)	–
Male sex of subjects in IL-33 studies, median (range), %	case: 45.91 (45.16–46.67) control: 47.22 (44.44–50)	–
Mean age of subjects in GGT studies, median (range), d*	case: 73.665 (62–112.5) control: 72.22 (57–75.3)	–
Male sex of subjects in GGT studies, median (range), %	case: 50.895 (25–54.29) control: 81.5 (58.8–82.86)	–
Mean age of subjects in APRi studies, median (range), d*	70.5 (58–83)	–
**Original nation**		
China	7 (58.33)	3726 (89.10)
USA	1 (8.33)	70 (1.67)
UK	1 (8.33)	260 (6.22)
Egypt	1 (8.33)	60 (1.43)
Japan	1 (8.33)	37 (0.88)
Mexico	1 (8.33)	29 (0.69)
**Test time**		
Before KPE	7 (58.33)	3655 (87.40)
Before/at KPE	1 (8.33)	70 (1.67)
At KPE	1 (8.33)	260 (6.22)
After KPE	2 (16.67)	160 (3.83)
Regardless of time	1 (8.33)	37 (0.88)

Abbreviations: MMP-7, matrix metallopeptidase-7; IL-33, interleukin 33; GGT, γ-glutamyl transferase; APRi, aspartate aminotransferase to platelet ratio index; M2BPGi, Mac-2-binding protein glycosylation isomer; KPE, Kasai portoenterostomy.

*Age of subjects is calculated from the day of birth to the day of sample collection.

### Biomarkers for differentiating BA from non-BA

**Figure 3:**
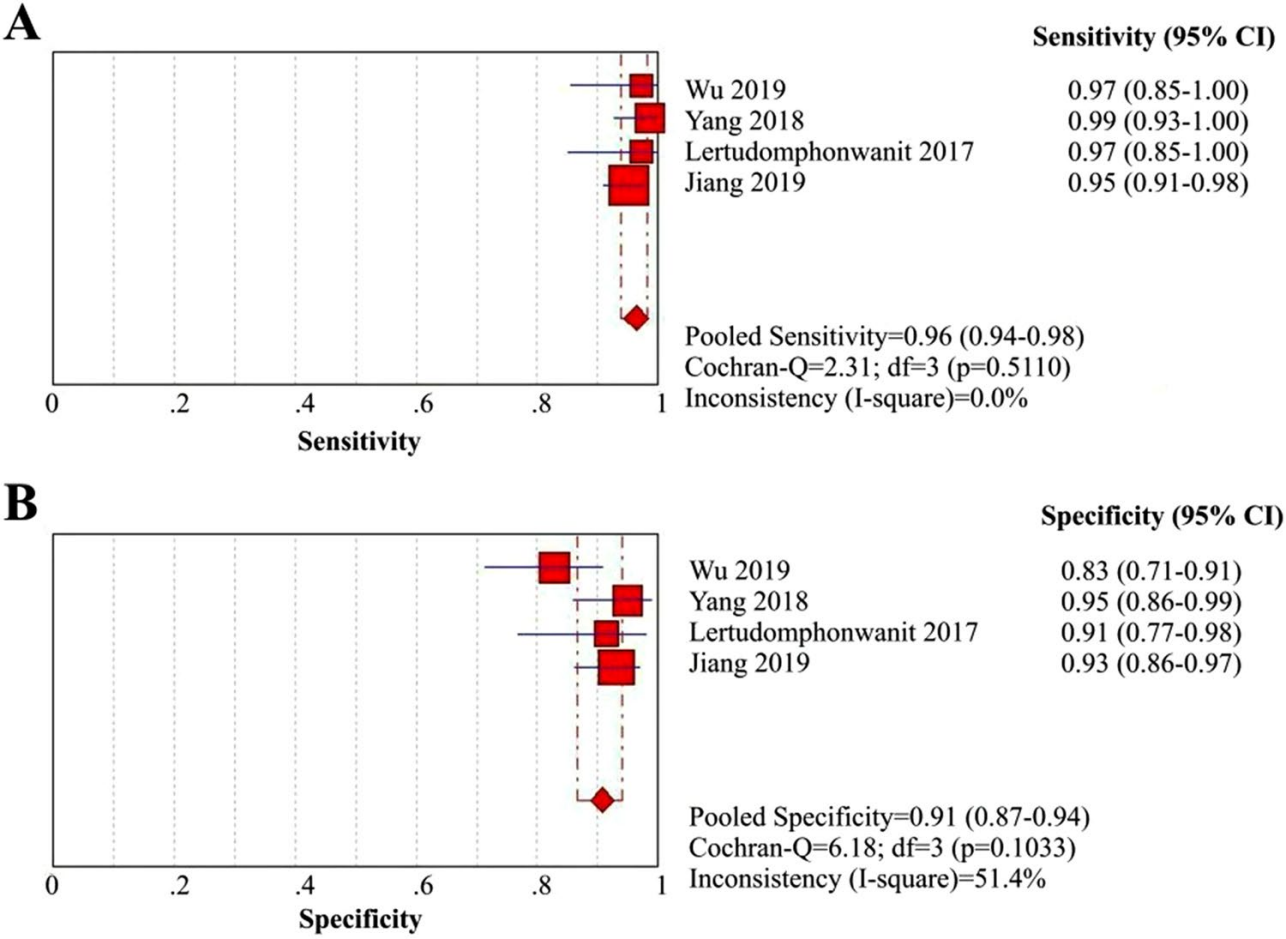
Coupled forest plots of the sensitivity and specificity of MMP-7 for BA diagnosis. (**A**) Sensitivity of MMP-7 for BA diagnosis; (**B**) Specificity of MMP-7 for BA diagnosis.

MMPs are key enzymes responsible for the degradation and deposition of all protein components in the extracellular matrix and basement membrane and participate in liver fibrosis caused by BA as well as other liver diseases^70^. Three retrospective cohort studies^57,58,60^ and one case–control study^59^ suggested that BA infants had a significantly higher level of serum MMP-7 than non-BA infants. Although the cutoff values used in these 4 articles were discordant, optimal diagnostic performance for serum MMP-7 in distinguishing BA from non-BA was demonstrated: the sensitivity, specificity and AUC ranged from 95 to 99%, 83% to 95% and 0.96 to 0.99, respectively (Table 3). The summary sensitivity and specificity of serum MMP-7were 96% (95% CI: 94–98%) and 91% (95% CI: 87–94%), respectively (Fig. 3A and 3B), and the PLR, NLR and DOR were 10.60 (95% CI: 5.48–20.52), 0.04 (95% CI: 0.02–0.07), and 313.42 (95% CI: 138.89–707.28), respectively (eFigure 1A, B and C *in Supplementary materials, page 4*). The AUC of MMP-7 for the diagnosis of BA was 0.9847 (eFigure 6A *in Supplementary materials, pages 9–10)*, indicating strong predictive accuracy.

**Table 3:** Summary of diagnostic test accuracy data in individual studies.

Study (Year)	Cut-off value	TP	FP	FN	TN	Sensitivity (%)	Specificity (%)	PLR	NLR	DOR	AUC	Ref
**MMP-7 for differentiating BA to non-BA**												
Wu (2019)	1.43 ng/mL	35	11	1	53	97	83	5.66	0.03	168.64	0.96	^57^
Yang (2018)	52.85 ng/mL	74	3	1	57	99	95	19.73	0.01	1,406.00	0.99	^58^
Lertudomphonwanit (2017)	NA	34	3	1	32	97	91	11.33	0.03	362.67	0.97	^59^
Jiang (2019)	10.37 ng/mL	178	7	9	94	95	93	13.73	0.05	265.59	0.98	^60^
**IL-33 for differentiating BA to non-BA**												
Behairy (2020)	20.8 pg/mL	29	1	1	29	94	97	28.06	0.07	420.5	0.995	^56^
Chen (2019)	314.1 pg/mL	19	10	12	35	61	78	2.76	0.5	5.54	0.67	^19^
**GGT for differentiating BA to non-BA**												
Lertudomphonwanit (2017)	300 U/L	24	6	5	25	83	77	3.64	0.23	16.15	0.9	^60^
Rendón-Macías (2008)	250 U/L	10	5	2	12	83	71	3.86	0.27	14.33	NA	^66^
Chen (2016)	303 IU/L	981	27	207	92	83	81	4.28	0.21	20	0.843	^69^
Dong (2018)	300 U/L	1188	44	324	172	79	80	2.83	0.24	12	0.845	^68^
**APRi for predicting post-KPE significant fibrosis**												
Yang (2015)	0.95	34	8	22	27	61	76	2.66	0.51	5.22	0.75	^63^
Ueno (2018)	0.88	13	2	8	14	62	88	4.95	0.44	11.38	0.88	^62^
**APRi for predicting post-KPE cirrhosis**												
Grieve (2013)	1.22	21	37	7	195	75	84	4.7	0.3	15.81	0.83	^61^
Yang (2015)	1.66	7	14	3	67	71	83	4.05	0.36	11.17	0.81	^62^
Ueno (2018)	0.88	10	5	1	21	91	81	4.73	0.11	42	0.88	^63^

Biomarker	Cases	Controls										
Diagnosed	TP	FP										
Undiagnosed	FN	TN										

Abbreviations: BA, biliary atresia; MMP-7, matrix metallopeptidase-7; IL-33, interleukin 33; APRi, aspartate aminotransferase to platelet ratio index; post-KPE, after Kasai portoenterostomy; TP, true positives; FP, false positives; FN, false negatives; TN, true negatives; PLR, positive likelihood ratio; NLR, negative likelihood ratio; DOR, diagnostic odds ratio; AUC, area under the curve.

Sensitivity = TP/Cases; Specificity = TN/Controls; PLR = Sensitivity/(1-Specificity); NLR = (1-Sensitivity)/Specificity; DOR = PLR/NLR.

**Figure 4:**
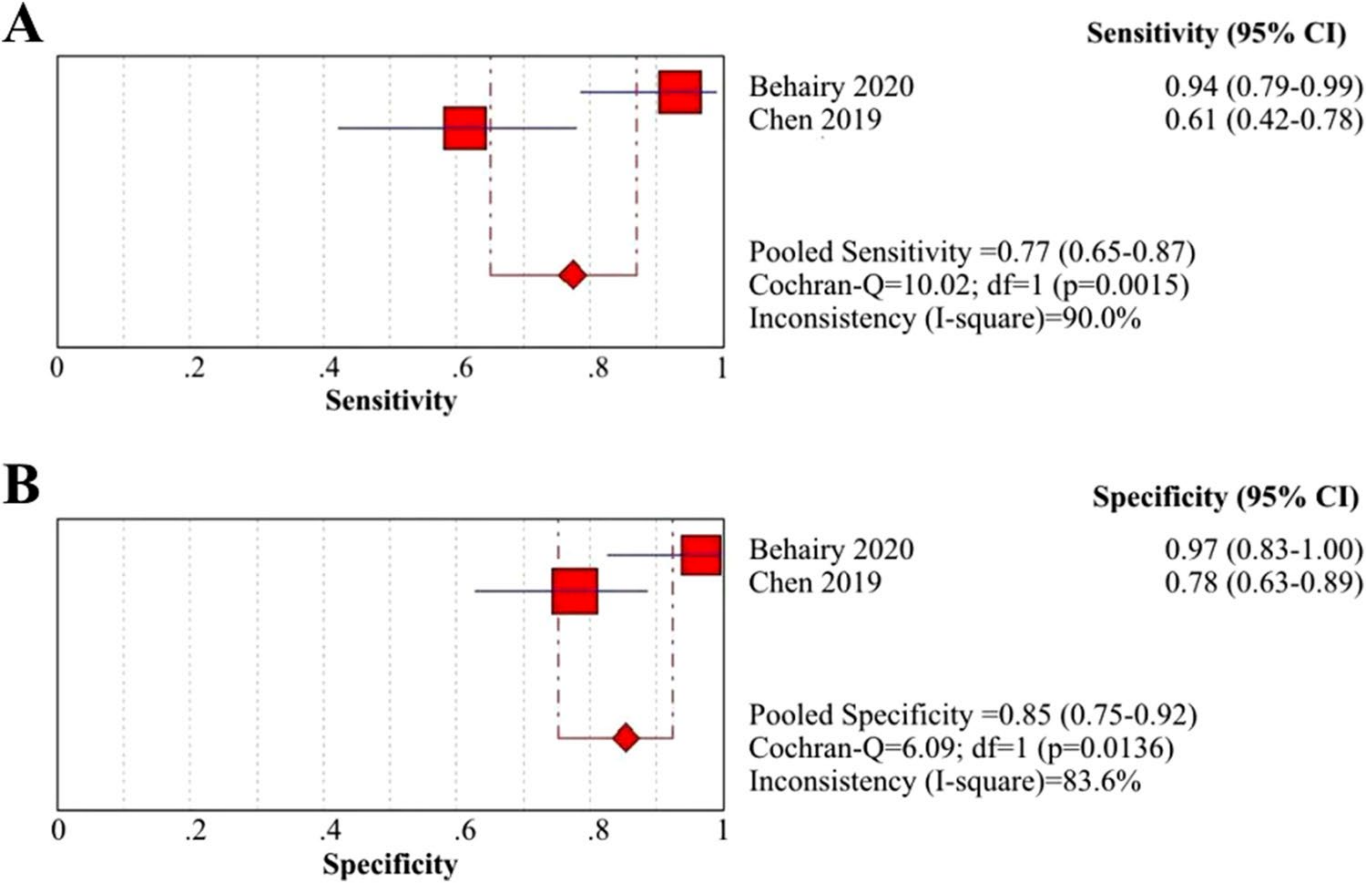
Coupled forest plots of the sensitivity and specificity of IL-33 for BA diagnosis. (**A**) Sensitivity of IL-33 for BA diagnosis; (**B**) Specificity of IL-33 for BA diagnosis.

One of the pivotal etiologies of BA is associated with inflammatory processes, whereby activated immune cells release proinflammatory cytokines that result in ongoing damage and obstruction of bile ducts and ductules^71^. Several case–control studies indicated that serum levels of IL-33 and IL-18 in BA patients were significantly greater than those in HC children^24–26,56^. We calculated the diagnostic performance of IL-33 for the identification of BA from non-BA using data from 2 of 9 selected articles^19,56^. With different cutoff values for each, the sensitivity, specificity and AUC of the included articles ranged from 61 to 94%, 78% to 97% and 0.67 to 0.995, respectively (Table 3). The summary sensitivity and specificity of IL-33 were 77% (95% CI: 65–87%) and 85% (95% CI: 75–92%) (Fig. 4A and B), respectively, and the PLR, NLR and DOR were 7.78 (95% CI: 0.48–127.48), 0.20 (95% CI: 0.02–1.90) and 41.76 (95% CI: 0.56–3,124.11), respectively (eFigure 2A, 2B and 2C *in Supplementary materials, page 5*).

**Figure 5:**
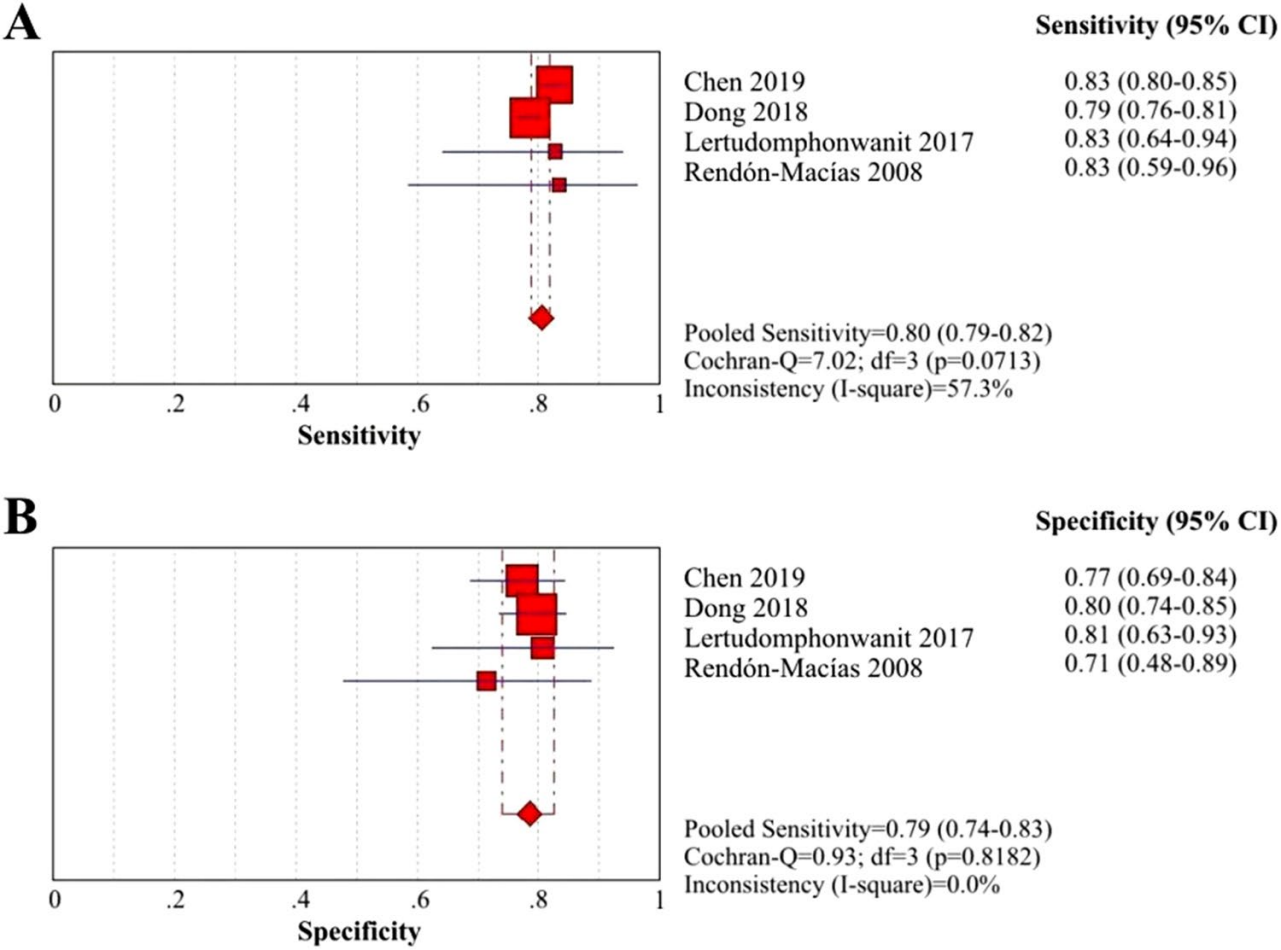
Coupled forest plots of the sensitivity and specificity of GGT for BA diagnosis. (**A**) Sensitivity of GGT for BA diagnosis; (**B**) Specificity of GGT for BA diagnosis.

GGT has been the most widely used biomarker for BA diagnosis in recent decades. Four retrospective cohort studies suggested that serum levels of GGT in BA patients were significantly higher than those in non-BA infants^59,66,68,69^, and they also provided data to calculate the diagnostic performance of GGT in the differentiation of BA from non-BA. The sensitivity, specificity and AUC of GGT ranged from 79 to 83%, 71% to 81% and 0.843 to 0.9, respectively, according to the cutoff value (Table 3). The summary sensitivity and specificity of GGT were 80% (95% CI: 79–82%) and 79% (95% CI: 74–83%) (Fig. 5A and B), respectively, and the PLR, NLR and DOR were 3.76 (95% CI: 3.09–4.57), 0.25 (95% CI: 0.23–0.28) and 15.06 (95% CI: 11.67–19.43), respectively (eFigure 3A, B and C *in Supplementary materials, pages 5–6*). The AUC of GGT for the diagnosis of BA was 0.9645 (eFigure 6B *in Supplementary materials, pages 9–10*), indicating great predictive accuracy.

### Biomarkers for post-KPE persistent jaundice

Similar to the inflammatory processes that trigger BA, inflammation also plays a key role in the postoperative period. Two case–control studies found that a postoperative increase in serum IL-18 levels was positively associated with post-KPE jaundice^25,26^. The findings suggest that serum IL-18 may serve as a marker for postoperative jaundice in BA patients.

### Biomarkers for post-KPE significant liver fibrosis

MMPs are not only involved in the pathogenesis of BA but also predict post-KPE clinical outcomes. Evidence from 4 case–control studies showed that a high serum concentration of preoperative MMP-7 correlates positively with the severity of post-KPE liver fibrosis^27,57,60,65^. Serum hyaluronic acid (HA) is a high-molecular-weight glycosaminoglycan that is produced and present in the extracellular matrix. Progressive liver diseases impair uptake of HA and raise the concentration of serum HA^72^. Elevated serum HA levels are a sensitive predictor of liver impairment. The results of some studies collectively suggest that the concentration of postoperative serum HA is associated with the severity of liver fibrosis^27–29^.

**Figure 6:**
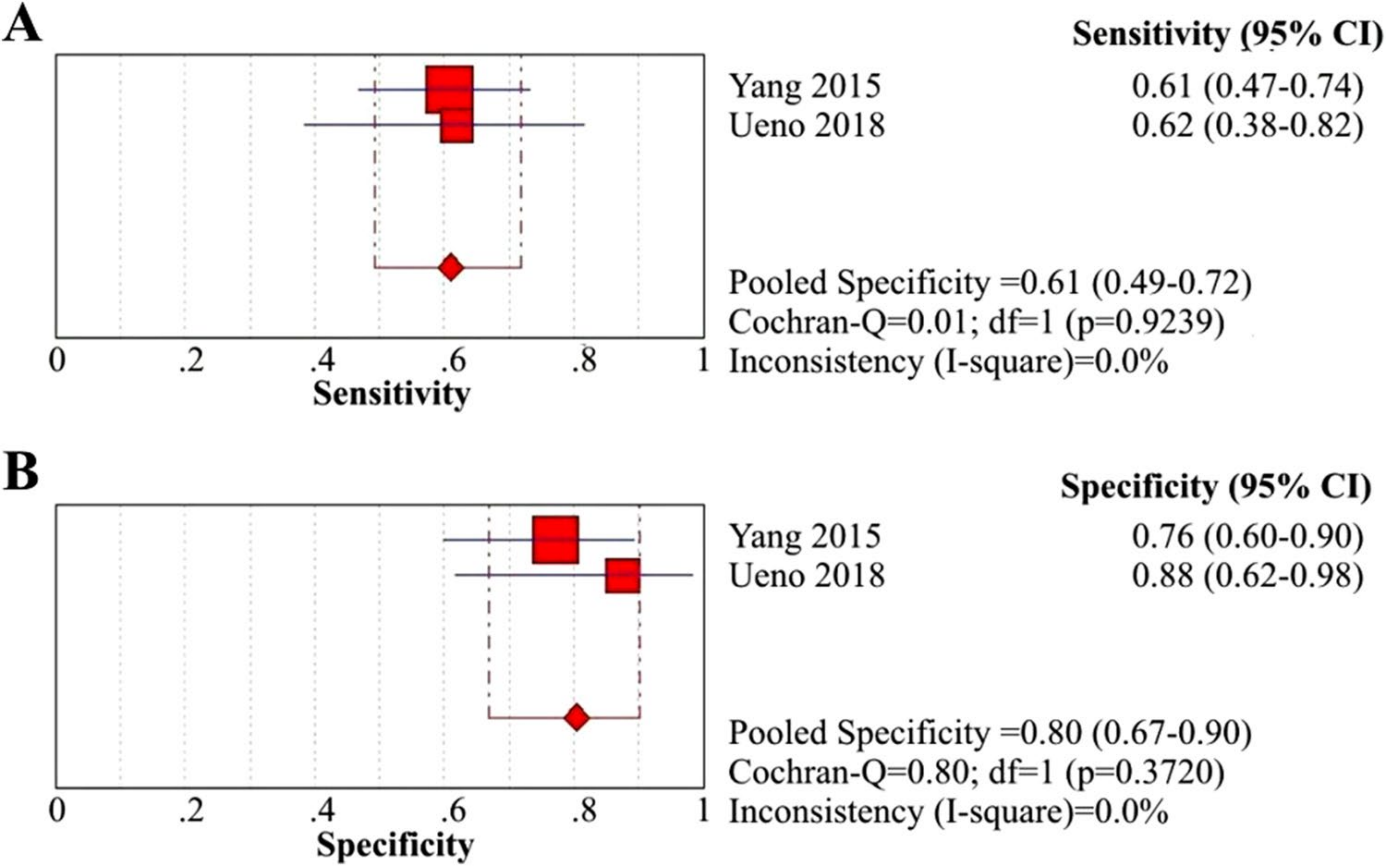
Coupled forest plots of the sensitivity and specificity of APRi for post-KPE significant liver fibrosis and cirrhosis. (**A**) Sensitivity of APRi for significant fibrosis; (**B**) Specificity of APRi for significant fibrosis.

Three case–control studies concluded that APRi was a sensitive biomarker for predicting post-KPE liver fibrosis^62,63,73^. We calculated the diagnostic performance of APRi in predicting post-KPE significant liver fibrosis from 2 of these 3 studies^62,63^. With different cutoff values of APRi, the sensitivity, specificity and AUC of the 2 included studies ranged from 61 to 62%, 76% to 88% and 0.75 to 0.88, respectively (Table 3). The summary sensitivity and specificity were 61% (95% CI: 49–72%) and 80% (95% CI: 67–90%), respectively (Fig. 6A and B), and the PLR, NLR and DOR were 3.09 (95% CI: 1.73–5.51), 0.49 (95% CI: 0.36–0.66) and 6.34 (95% CI: 2.89–13.90), respectively (eFigure 4A, B and C *in Supplementary materials, pages 6–7*).

### Biomarkers for post-KPE cirrhosis

**Figure 7:**
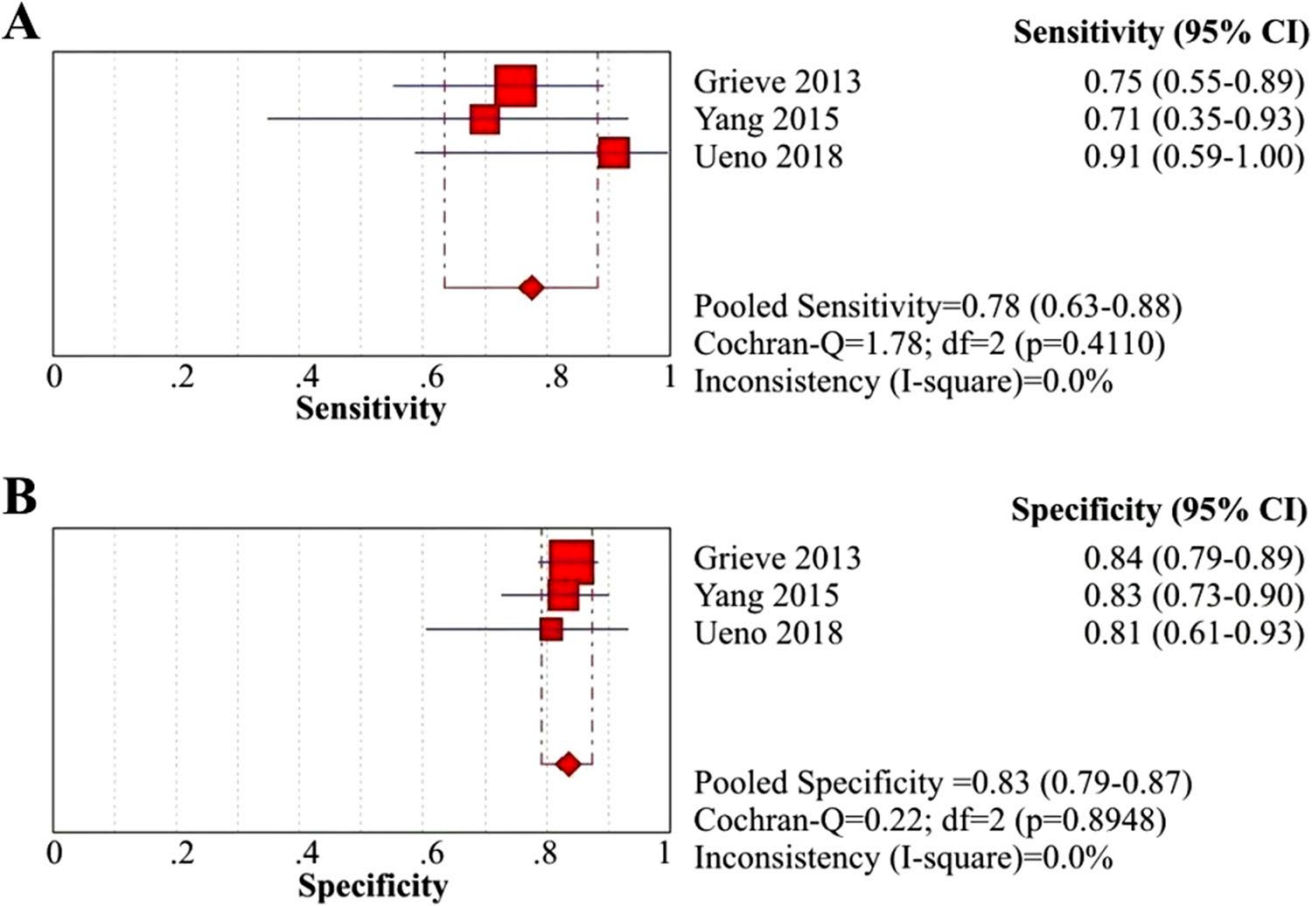
Coupled forest plots of the sensitivity and specificity of APRi for post-KPE cirrhosis. (**A**) Sensitivity of APRi for cirrhosis; (**B**) Specificity of APRi for cirrhosis.

The progression of liver fibrosis eventually leads to the occurrence of cirrhosis, for which liver transplantation is required as the only solution. Discovery of noninvasive methods to predict the occurrence of cirrhosis would enable effective prevention of the development of liver failure and the need for liver transplantation in BA patients. Three of our 9 included studies found that APRi was a useful noninvasive tool to predict post-KPE cirrhosis^61–63^. The sensitivity, specificity and AUC of APRi with different cutoff values in these studies ranged from 71 to 91%, 81% to 84% and 0.81 to 0.88, respectively (Table 3). The summary sensitivity and specificity were 78% (95% CI: 63–88%) and 83% (95% CI: 79–87%), respectively (Fig. 7A and B); PLR, NLR and DOR were 4.56 (95% CI:3.37–6.18), 0.28 (95% CI: 0.17–0.46) and 16.69 (95% CI: 8.34–33.38), respectively (eFigure 5A, B and C *in Supplementary materials, pages 7–8*). The AUC of APRi for the prediction of post-KPE cirrhosis was 0.8729 (eFigure 6C *in Supplementary materials, pages 9–10*), indicating high predictive accuracy.

M2BPGi, also known as *Wisteria floribunda* agglutinin-positive human Mac-2-binding protein, has been used as a glycol biomarker of liver fibrosis in patients with chronic hepatitis C^74^. Recently, two studies from Japan reported that M2BPGi was capable of predicting post-KPE cirrhosis in BA^63,64^.

### Heterogenicity and threshold effect

Most analyses did not find significant heterogenicity based on Cochran’s Q test and Higgins I^2^ statistic test, whereas the following analyses showed substantial to considerable heterogenicity, as follows: (1) PLR (*p* = 0.0499, I^2^ = 61.0%) of serum MMP-7 in BA diagnosis; (2) sensitivity (*p* = 0.0015, I^2^ = 90.0%), specificity (*p* = 0.0136, I^2^ = 83.6%), PLR (*p* = 0.0055, I^2^ = 87.0%), NLR (*p* = 0.0014, I^2^ = 90.2%) and DOR (*p* = 0.0011, I^2^ = 90.6%) of serum IL-33 in BA diagnosis (eTable 2in *Supplementary materials, page 3*). The Spearman correlation coefficient revealed no threshold effects in the analyses of MMP-7 for BA diagnosis or APRi for predicting cirrhosis, and the weighted regression of their SROC curves showed no heterogeneity (eTable 3 in *Supplementary materials, page 3*).

## Discussion

The existing diagnostic methods of BA diagnosis rely on invasive procedures such as surgical exploration and operative cholangiogram, and all preoperative tests are unreliable. Although the Kasai operation offers potential bile drainage, it has a narrow treatment window, and a large proportion of patients experience ongoing problems, including persistent cholestasis, portal hypertension, liver fibrosis and cirrhosis. The monitoring of persistent jaundice requires continued measurements of total bilirubin, while liver fibrosis is traditionally evaluated by liver biopsy. Therefore, noninvasive measures that promote early diagnosis and recognition of complications are beneficial. Serum biomarkers as a screening or prognostic tool have been widely used for other conditions, such as degenerative and malignant diseases^75,76^. Various biomarkers for BA have been reported, but there is a lack of high-level evidence to confirm their values. Herein, we conduct a systematic review and meta-analysis that investigates the most appropriate noninvasive biomarkers for diagnosing BA and predicting post-KPE outcomes.

Inflammation is a trigger factor that can cause an autoimmune response against antigens from the biliary epithelium^77^. Immune-mediated biliary injury is characterized by overexpression of histocompatibility antigens on bile ducts and obvious infiltration of immunologically active T lymphocytes, which is a central feature of adult liver diseases and is also related to obstruction of neonatal extrahepatic bile ducts. Our study confirms the diagnostic performance of serum IL-33 in the early diagnosis of BA and provides good evidence that IL-18 can predict post-KPE persistent jaundice. IL-33 and IL-18 are the two most closely related and best-characterized members of the IL-1 family^78^. Dong et al.^24^ found that the serum IL-33 level was significantly higher in BA patients (791.0 ± 22.22 pg/mL) than in non-BA (607.1 ± 20.68 pg/mL) and HC (588.5 ± 27.71 pg/mL) groups (both *p* < 0.001), but no significant difference was observed between the latter groups (*p* > 0.05). However, one study by Vejchapipat and colleagues^25^ found elevated serum IL-18 in medium-term BA survivors, which increased significantly with the severity of post-KPE persistent jaundice (*p* = 0.004). Taken together, inflammatory factors and the autoimmune response are both involved in the etiology and disease progression of BA.

The diagnostic performance of serum MMP-7 in the early diagnosis of BA and its good level of evidence for predicting post-KPE significant liver fibrosis are presented here. Of note, the collection of serum MMP-7 was performed prior to the implementation of KPE. Overall, serum MMP-7 is a reliable biomarker to diagnose BA in a clinical setting due to its high specificity (95–99%) and sensitivity (83–95%)^57–60^. γ-Glutamyltransferase (GGT), one of the factors measured in biochemical liver function tests, is also utilized to differentiate BA from non-BA; at a cutoff value of 250-303 IU/L, the sensitivity and specificity were 82.8–83.3% and 70.6–81.6%, respectively^59,66,69^, suggesting that the diagnostic accuracy of MMP-7 is significantly higher than that of GGT. In addition, Wu et al.^57^ showed a positive correlation between serum MMP-7 and the severity of liver fibrosis in infants with cholestasis at a mean age of 1.5 months, indicating that MMP-7 is likely useful for predicting post-KPE liver fibrosis in young BA patients. Furthermore, the MMP-7 protein was found to be significantly elevated in BA patients with persistent cholestasis and liver fibrosis^79–81^. Therefore, MMP-7 is a very valuable biomarker for both BA diagnosis and post-KPE prognosis.

Although there are no biomarkers with good evidence levels for post-KPE portal hypertension, we found that serum HA and APRi to have good evidence levels in association with post-KPE liver fibrosis. HA, a linear polysaccharide, is responsible for liver fibrogenesis; hepatic production of HA is predominantly carried out by hepatic stellate and myofibroblast-like cells^82^. The serum HA level in HC children is low, whereas it is significantly increased in BA patients^34^, suggesting that HA is a potential biomarker for differentiating BA from HC. Additionally, the concentration of serum HA correlates positively with the severity of post-KPE cirrhosis and its complications in BA patients^28^, such as ascites or esophageal varices, both of which reflect the clinical characteristics of portal hypertension.

APRi was first introduced as a noninvasive tool by Wai and colleagues in 2003 to predict significant liver fibrosis and cirrhosis in adults with chronic hepatitis C^83^, and it has been employed as a biomarker in the evaluation of liver fibrosis in BA patients^61,84^. This measure is calculated as serum aspartate aminotransferase level (U/L)/upper normal × 100/platelet count (10^3^/μL)^83^. Our current meta-analysis revealed a high diagnostic accuracy of APRi for predicting post-KPE significant liver fibrosis and cirrhosis. Early reports suggested that postoperative APRi might help predict post-KPE esophageal varices and native liver survival in BA patient^85,86^. Nevertheless, it is worth mentioning that no correlation between preoperative APRI and native liver survival has been observed^67^. One study by Yang et al.^62^ further showed that preoperative APRI correlated significantly with post-KPE metavir scores (a scoring system to quantify liver fibrosis) in BA patients and could predict post-KPE persistent jaundice and cirrhosis, despite the different reference values among centers. In the clinical setting, these results should be interpreted with other clinical parameters. Similarly, serum M2BPGi has been confirmed as a novel biomarker for predicting post-KPE cirrhosis in BA patients; its AUC (0.93) is higher than that of APRi (0.81) and the fibrosis-4 index (FIB-4) (0.59)^63,64^. A study by Kuno^74^ found that serum M2BPGi was also a sensitive biomarker for predicting significant liver fibrosis and cirrhosis in adults with chronic viral hepatitis, and the performance for cirrhosis prediction was superior to that of FIB-4 and HA. Our present study consistently demonstrates a high diagnostic accuracy of M2BPGi in the prediction of post-KPE cirrhosis in BA patients.

**Figure 8:**
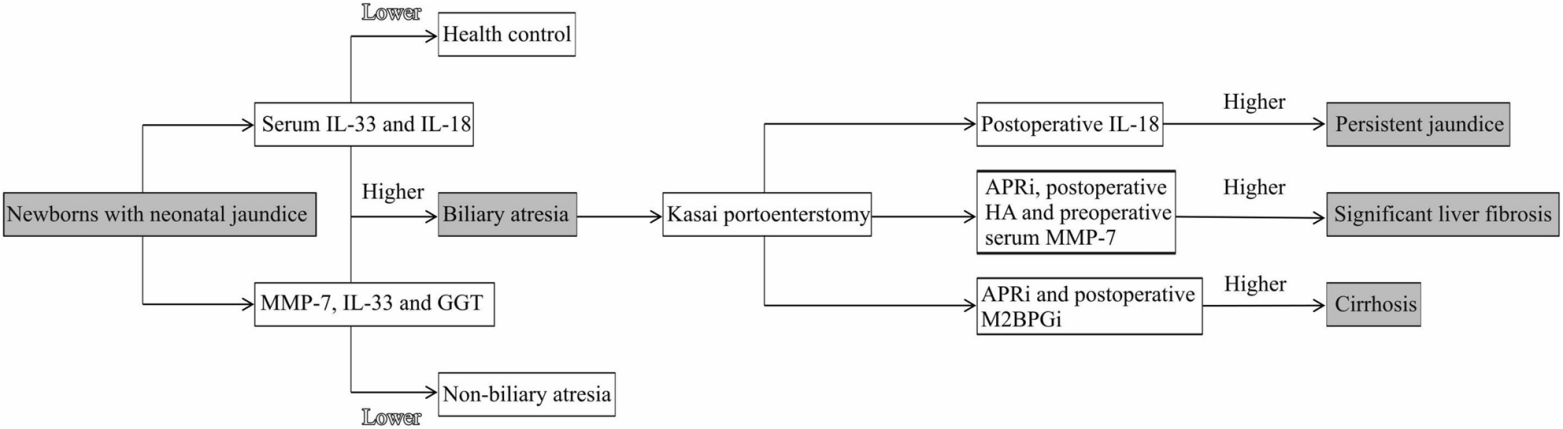
Flow diagram of practical strategy using biomarkers for diagnosing BA and prognosing post-KPE clinical outcomes. Abbreviations: MMP-7, matrix metallopeptidase-7; IL, interleukin; GGT, γ-glutamyl transferase; HA, hyaluronic acid; APRi, aspartate aminotransferase to platelet ratio index; M2BPGi, Mac-2-binding protein glycosylation isomer. Selecting the best cut-off value is based on the largest AUC. The cut-off value is not presented in the investigations of serum IL-18 for diagnosing BA and prognosing post-KPE persistent jaundice and serum HA for prognosing post-KPE significant fibrosis. The best cut-off values of MMP-7 for diagnosing BA and prognosing post-KPE significant liver fibrosis both are 52.85 ng/mL with the largest AUC of 0.99^58^; the best cut-off value of IL-33 for diagnosing BA is 20.8 pg/mL with the largest AUC of 0.995^56^; the best cut-off value of GGT for diagnosing BA is 300 U/L with the largest AUC of 0.9^60^; the best cut-off value of APRi for prognosing liver fibrosis and cirrhosis is 0.88 with the largest AUC of 0.88^62^.

Based on our analysis, we propose using biomarkers to assist in the diagnosis of BA and in the monitoring of postoperative outcomes (Fig. 8). The best cutoff value of biomarkers, if obtainable, can be selected in terms of the largest AUC value. A high concentration of serum IL-33 or IL-18 distinguishes newborns with BA from HCs, and differentiation of BA from non-BA can be suggested by measuring levels of serum MMP-7, IL-33 and GGT. Regarding the prediction of post-KPE prognosis, a higher serum IL-18 level indicates the occurrence of persistent jaundice; an increased value of APRi, preoperative serum MMP-7 level or postoperative serum HA level predicts the occurrence of significant liver fibrosis. A value of APRi or postoperative serum M2BPGi higher than the best cutoff value suggests liver cirrhosis.

Nonetheless, biomarkers are not without limitations. The universal use of biomarkers for BA screening may increase the health-care budget; on the other hand, it might minimize the socioeconomic burden on BA by improving outcomes. Furthermore, such an approach might replace other screening policies, such as stool color cards, as already practiced in mainland China, Japan and Taiwan^87^. In general, the clinical application of some biomarkers is limited by availability. Last, the best cutoff value of some of the biomarkers, including MMP-7, IL-33 and IL-18, remained unclear, and further study will be required to determine their cutoff values.

We acknowledge a number of limitations in our study. Our meta-analysis included all currently available relevant English-language literature, but publication bias may exist due to the small number of papers analyzed. In addition, partial analyses detected significant heterogeneity, which might lead to further bias. Only 12 studies have the complete data available for meta-analysis and the others could be included for systematic review only. Moreover, because only a few publications were included in the analysis of BA diagnosis based on serum IL-33 and prediction of post-KPE significant liver fibrosis with APRi, the threshold effect and SROC curves were not obtained in either analysis. Last, the present work was a diagnostic meta-analysis, and all the included studies were either case–control or cohort studies rather than randomized controlled trials, which limited the calculation of predictive values and the evidence level of the biomarkers studied.

## Conclusions

Serum IL-33 and IL-18 are both useful biomarkers for differentiating BA from HC, and serum MMP-7, IL-33 and GGT are applicable biomarkers to distinguish BA from non-BA. After KPE, biomarkers predicting the prognosis may include (1) the serum IL-18 level to predict persistent jaundice; (2) APRi, postoperative serum HA and preoperative serum MMP-7 to predict significant liver fibrosis; and (3) APRi and postoperative serum M2BPGi to predict cirrhosis. These noninvasive biomarkers should be incorporated into the management strategy for BA.

## Supplementary Information

Supplementary Information.
