# Supplementary material for: Biomarkers for the diagnosis and post-Kasai portoenterostomy prognosis of biliary atresia: a systematic review and meta-analysis
Source: Sci Rep. 2021 Jun 3;11:11692. doi: 10.1038/s41598-021-91072-y (PMC8175424; doi:10.1038/s41598-021-91072-y)
Supplement: Supplementary file 1 — Supplementary Information. [file 41598_2021_91072_MOESM1_ESM.doc]

| **Original position** | **Biomarker** | **BA vs HC** | **BA vs non-BA** | **post-KPE persistent jaundice** | **post-KPE portal hypertension** | **post-KPE significant liver fibrosis** |
| --- | --- | --- | --- | --- | --- | --- |
| Serum | MIP-3α |  | ↑ |  |  |  |
|  | MMP-3 | ↑ |  | ⊕ | ⊕ |  |
|  | MMP-7 | ↑ | ↑ |  |  | ⊕ |
|  | IL-8 | ↑ |  |  |  |  |
|  | IL-12p40 |  |  | ∅ |  |  |
|  | IL-18 | ↑ |  | ⊕ |  |  |
|  | IL-33 | ↑ |  |  |  |  |
|  | IFN-γ | ↑ |  |  |  |  |
|  | FGF-21 |  | ↑ |  |  | ⊕ |
|  | anti-BPO |  | ↑ |  |  |  |
|  | ANAs |  | ↑ |  |  |  |
|  | UDP-IV |  | ↑ |  |  |  |
|  | Apo C-II | ↓ | ↑ |  |  |  |
|  | Apo C-III | ↑ | ↑ |  |  |  |
|  | APRi |  |  |  |  | ⊕ |
|  | HMGB1 | ↑ | ↑ |  |  |  |
|  | HA |  | ↑ |  |  | ⊕ |
|  | adiponectin | ↑ |  | ⊕ |  | ⊕ |
|  | galectin-3 | ↑ |  | ⊕ | ⊕ | ⊕ |
|  | TIMP-1 | ↑ |  | ⊕ | ⊕ |  |
|  | bFGF | ↑ |  | ⊕ | ⊕ |  |
|  | HSP70 | ↑ |  | ⊕ | ⊕ |  |
|  | periostin | ↑ |  | ⊕ |  | ⊕ |
|  | sRAGE | ↑ |  | ⊕ |  | ⊕ |
|  | ANCAs | ↑ |  |  |  |  |
|  | sICAM-1 | ↑ |  |  |  |  |
|  | GPC3 |  | ↑ | ⊕ |  | ⊕ |
|  | GATA6 | ↑ |  |  |  |  |
|  | GGT |  | ↑ |  |  | ⊕ |
|  | uPAR | ↑ |  | ⊕ |  | ⊕ |
|  | ATX | ↑ |  |  |  |  |
|  | TGF-β1 | ↑ |  | ⊕ |  |  |
|  | RBP4 | ↓ |  | ∅ | ∅ | ∅ |
|  | 25-(OH)D |  |  | ⊕ |  |  |
|  | M2BPGi |  |  | ⊕ |  |  |
|  | miR-4689 | ↑ |  |  |  |  |
|  | miR-200b/429 |  | ↑ |  |  |  |
|  | miR-4429 | ↓ |  |  |  |  |
|  | miR-140-3p | ↓ |  |  |  |  |
| Plasma | ET-1 |  | ↑ |  | ⊕ | ⊕ |
|  | endoglin |  | ↑ | ⊕ | ⊕ |  |
|  | 8-OHdG | ↑ |  |  |  |  |
|  | TCDCA/CDCA | ↑ | ↑ |  |  |  |
| Leukocyte | mtDNA copy number | ↓ |  |  |  | ∅ |
|  | RTL | ↓ |  | ∅ |  | ∅ |

**eTable 1. Summary of biomarkers in identifications of biliary atresia diagnosis and post-KPE prognosis.**

↑ in green square and ↓ in yellow square mean significant higher and significant lower respectively.

⊕ in turquoise square and ∅ in red mean positive correlation and negative correlation respectively.

Abbreviations: BA, biliary atresia; HC, health control; MIP-3α, macrophage inflammatory protein-3α; FGF-21, fibroblast growth factor 21; ANAs, anti-nuclear antibodies; UDP-IV, urinary dipeptidyl peptidase IV; Apo, apolipoprotein; HMGB1, High Mobility Group Box 1; HA, hyaluronic acid; TIMP-1, tissue inhibitors of metalloproteinase 1; bFGF, basic fibroblast growth factor; HSP70, heat shock protein 70; sRAGE, soluble receptor for advanced glycation end products; ANCAs, antineutrophil cytoplasmic antibodies; sICAM-1, soluble intercellular adhesion molecule-1; GPC3, glypican-3; GGT, γ-glutamyltransferase; uPAR, urokinase plasminogen activator receptor; ATX, autotaxin; TGF-β1, transforming growth factor-β1; RBP4, retinol binding protein 4; M2BPGi, Mac-2 binding protein glycosylation-modified isomer; ET-1, endothelin-1; 8-OHdG, 8-hydroxy-2'-deoxyguanosine; TCDCA/CDCA, The ratio of taurochenodeoxycholic acid level to chenodeoxycholic acid level; mtDNA, mitochondrial DNA; RTL, relative telomere length.

| **Analysis** | **Cochran Q (*p*)** | | | | | **Higgins I^2^** | | | | | |  |
| --- | --- | --- | --- | --- | --- | --- | --- | --- | --- | --- | --- | --- |
|  | **Sensitivity** | **Specificity** | **PLR** | **NLR** | **DOR** | | **Sensitivity** | **Specificity** | **PLR** | **NLR** | **DOR** | |
| MMP-7 for BA diagnosis | 0.5110 | 0.1033 | 0.0499 | 0.5656 | 0.5508 | | 0.0% | 51.4% | 61.0% | 0.0% | 0.0% | |
| IL-33 for BA diagnosis | 0.0015 | 0.0136 | 0.0055 | 0.0014 | 0.0011 | | 90.0% | 83.6% | 87.0% | 90.2% | 90.6% | |
| GGT for BA diagnosis | 0.0710 | 0.8180 | 0.8770 | 0.3470 | 0.9400 | | 57.3% | 0.0% | 0.0% | 9.2% | 0.0% | |
| APRi for predicting significant liver fibrosis | 0.9239 | 0.3720 | 0.4070 | 0.6540 | 0.4376 | | 0.0% | 0.0% | 0.0% | 0.0% | 0.0% | |
| APRi for predicting cirrhosis | 0.4110 | 0.8948 | 0.9710 | 0.5399 | 0.6275 | | 0.0% | 0.0% | 0.0% | 0.0% | 0.0% | |

**eTable 2. Heterogenicity test.**

| **Analysis** | **Spearman correlation coefficient** | **Weighted regression *p* value** |
| --- | --- | --- |
| MMP-7 for BA diagnosis | -0.200 (*p=*0.800) | 0.876 |
| GGT for BA diagnosis | 0.400 (*p*=0.600) | 0.820 |
| APRi for cirrhosis of BA after KPE | 0.500 (*p*=0.667) | 0.525 |

**eTable 3. Diagnostic threshold effect and weighted regression.**

**eFigure 1**. **Coupled forest plots of the positive likelihood ratio, negative likelihood ratio and diagnostic odds ratio of MMP-7 for biliary atresia diagnosis.**

**
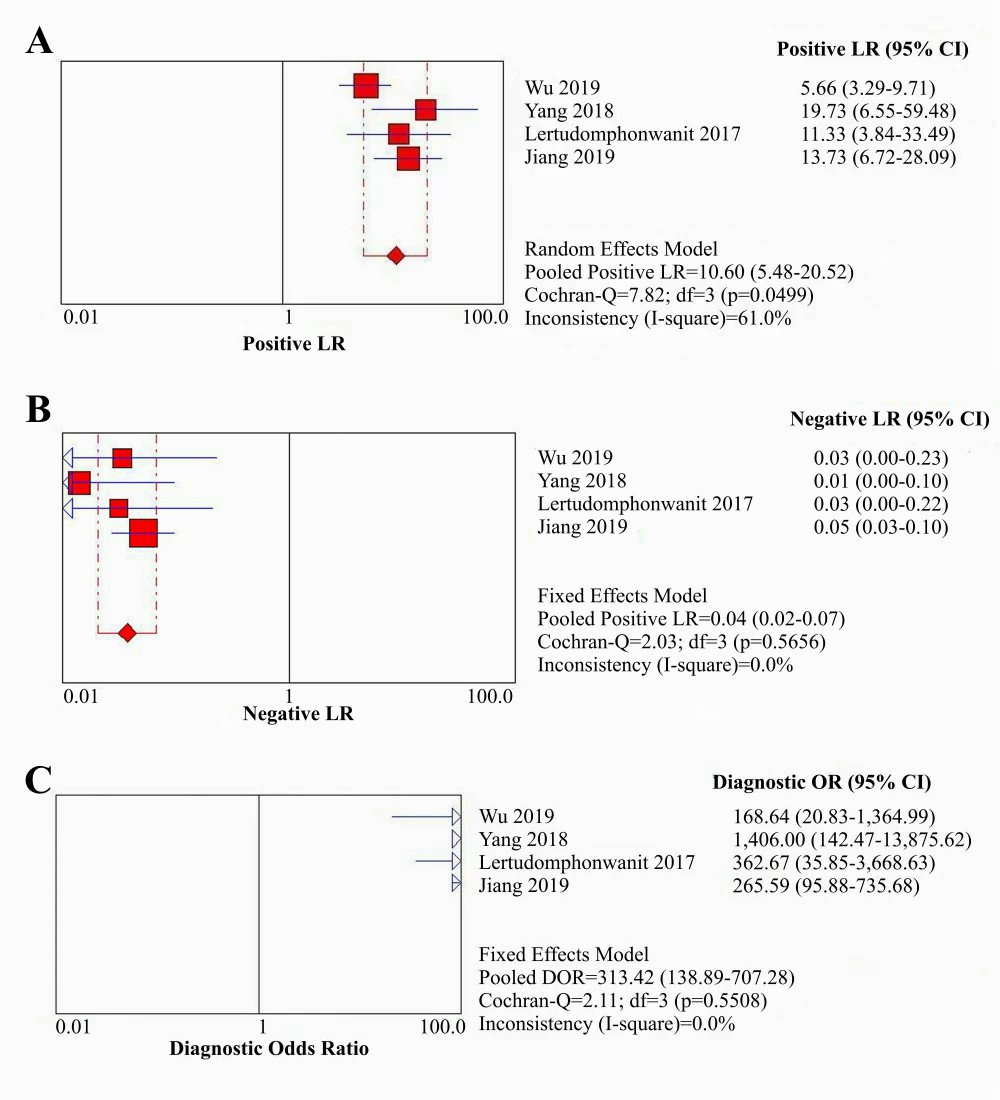
**

A. Positive likelihood ratio of MMP-7 for BA diagnosis; B. Negative likelihood ratio of MMP-7 for BA diagnosis; C. Diagnostic odds ratio of MMP-7 for BA diagnosis.

Abbreviations: LR, likelihood ratio; OR, odds ratio; DOR, diagnostic odds ratio.

**eFigure 2**. **Coupled forest plots of the positive likelihood ratio, negative likelihood ratio and diagnostic odds ratio of IL-33 for biliary atresia diagnosis.**

**
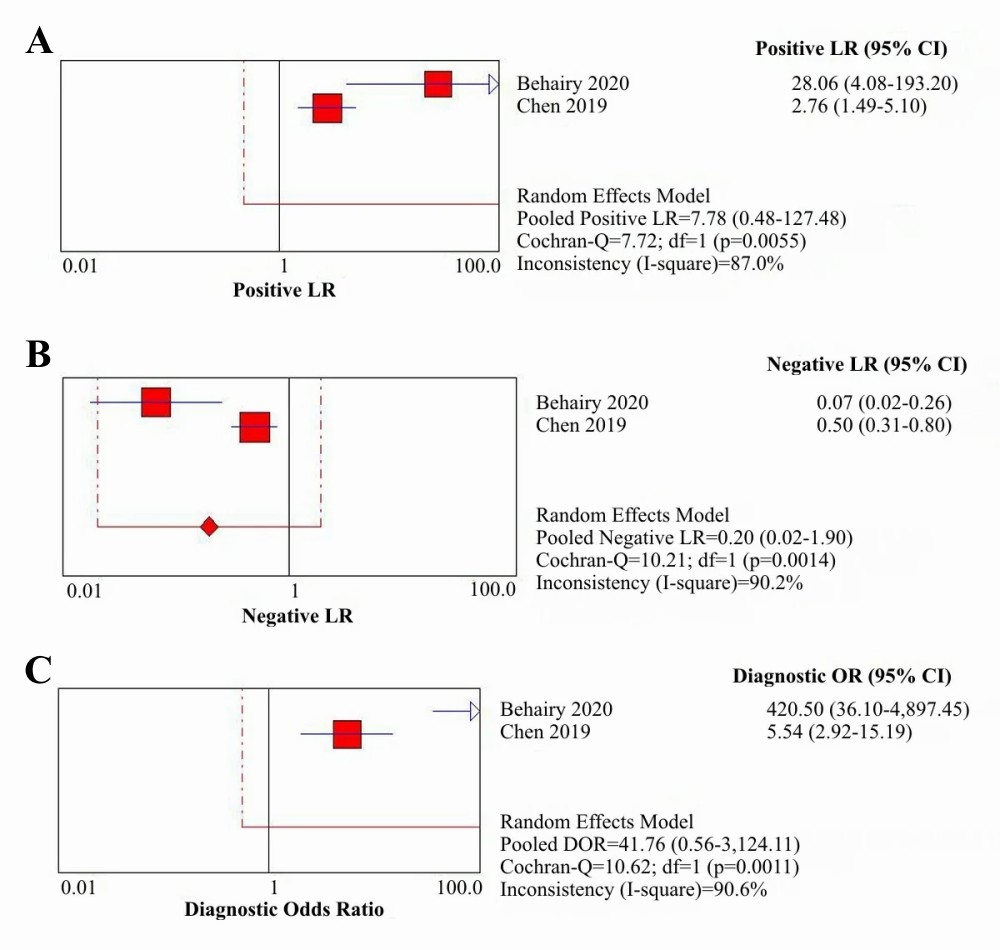
**

A. Positive likelihood ratio of IL-33 for BA diagnosis; B. Negative likelihood ratio of IL-33 for BA diagnosis; C. Diagnostic odds ratio of IL-33 for BA diagnosis.

Abbreviations: LR, likelihood ratio; OR, odds ratio; DOR, diagnostic odds ratio.

**eFigure 3**. **Coupled forest plots of the positive likelihood ratio, negative likelihood ratio and diagnostic odds ratio of GGT for biliary atresia diagnosis.**

**
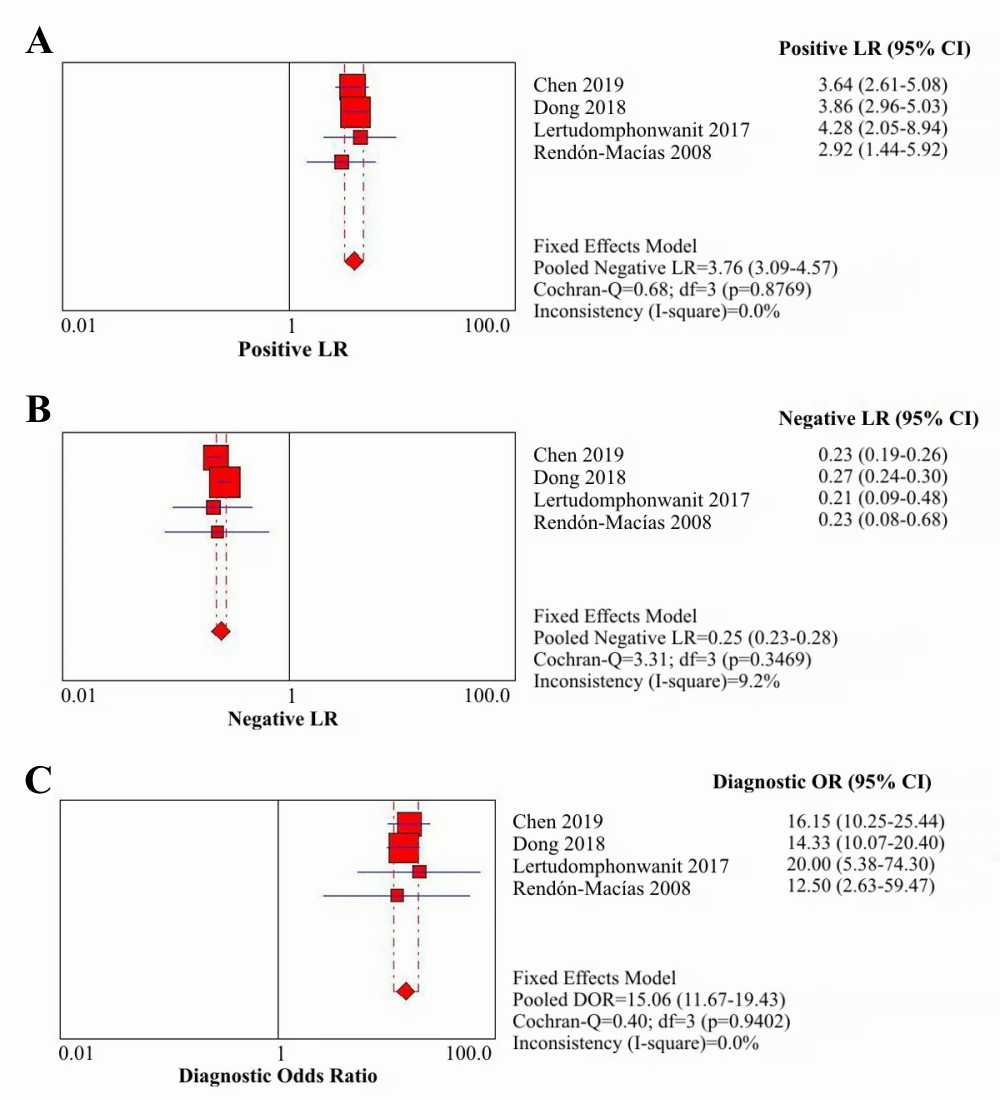
**

A. Positive likelihood ratio of GGT for BA diagnosis; B. Negative likelihood ratio of GGT for BA diagnosis; C. Diagnostic odds ratio of GGT for BA diagnosis.

Abbreviations: LR, likelihood ratio; OR, odds ratio; DOR, diagnostic odds ratio.

**eFigure 4**. **Coupled forest plots of the positive likelihood ratio, negative likelihood ratio and diagnostic odds ratio of APRi for post-KPE significant fibrosis of BA patients.**

**
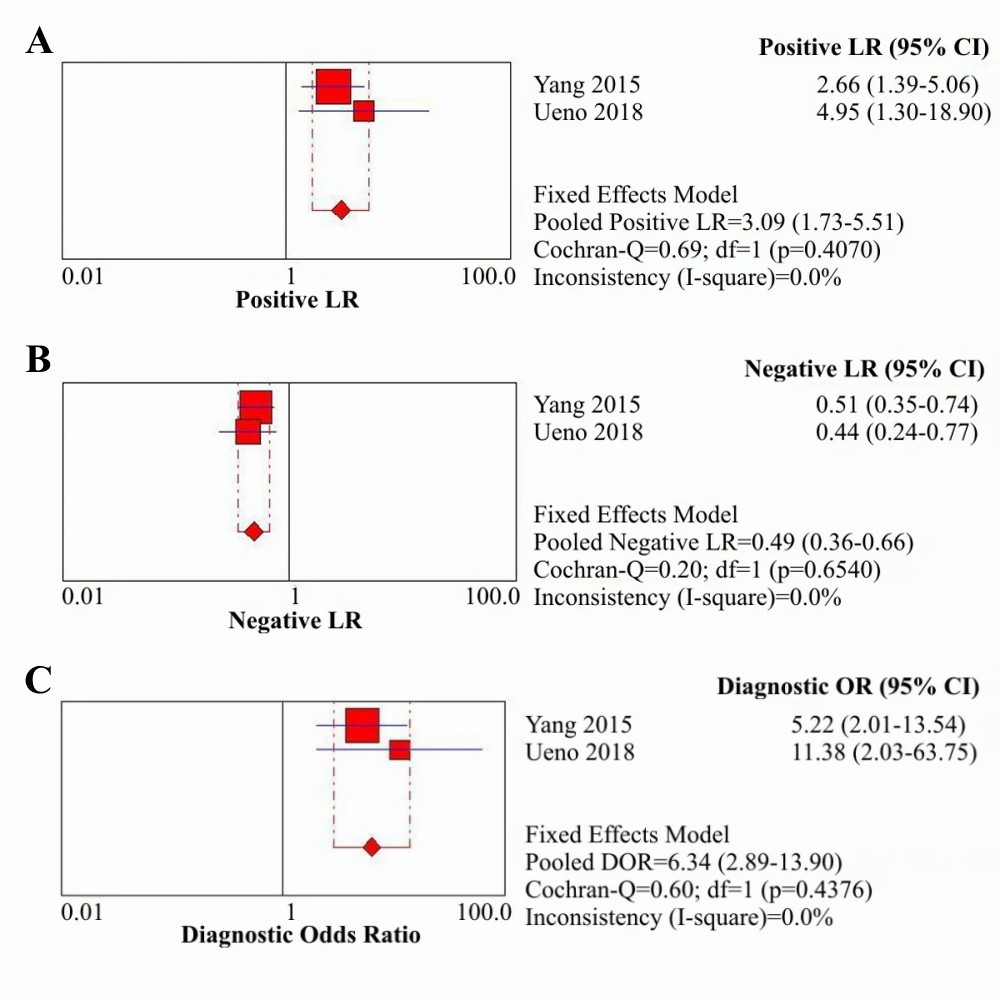
**

A. Positive likelihood ratio of APRi for significant fibrosis; B. Negative likelihood ratio of APRi for significant fibrosis; C. Diagnostic odds ratio of APRi for significant fibrosis.

Abbreviations: LR, likelihood ratio; OR, odds ratio; DOR, diagnostic odds ratio.

**eFigure 5**. **Coupled forest plots of the positive likelihood ratio, negative likelihood ratio and diagnostic odds ratio of APRi for post-KPE cirrhosis** **of BA patients.**

**
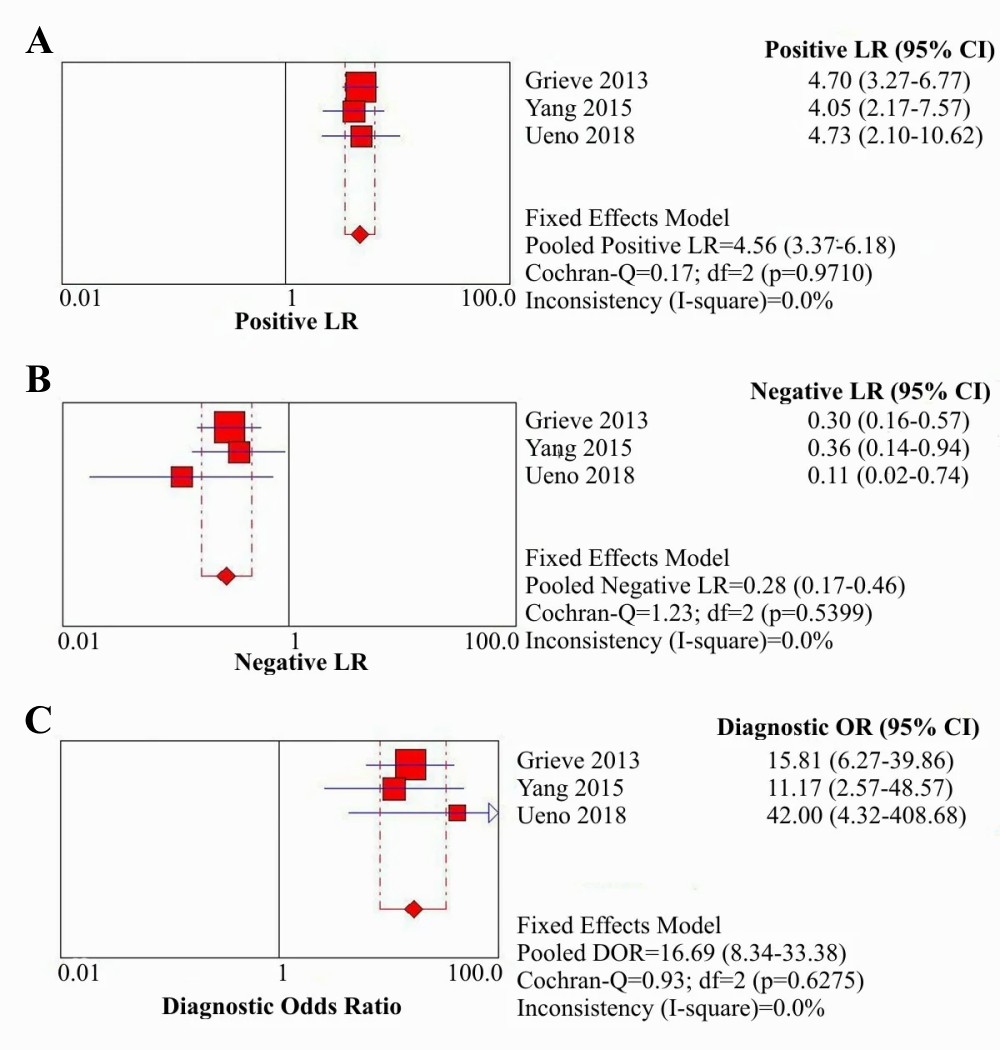
**

A. Positive likelihood ratio of APRi for cirrhosis; B. Negative likelihood ratio of APRi for cirrhosis; C. Diagnostic odds ratio of APRi for cirrhosis.

Abbreviations: LR, likelihood ratio; OR, odds ratio; DOR, diagnostic odds ratio.

**eFigure 6. The analysis of summary ROC curve.**


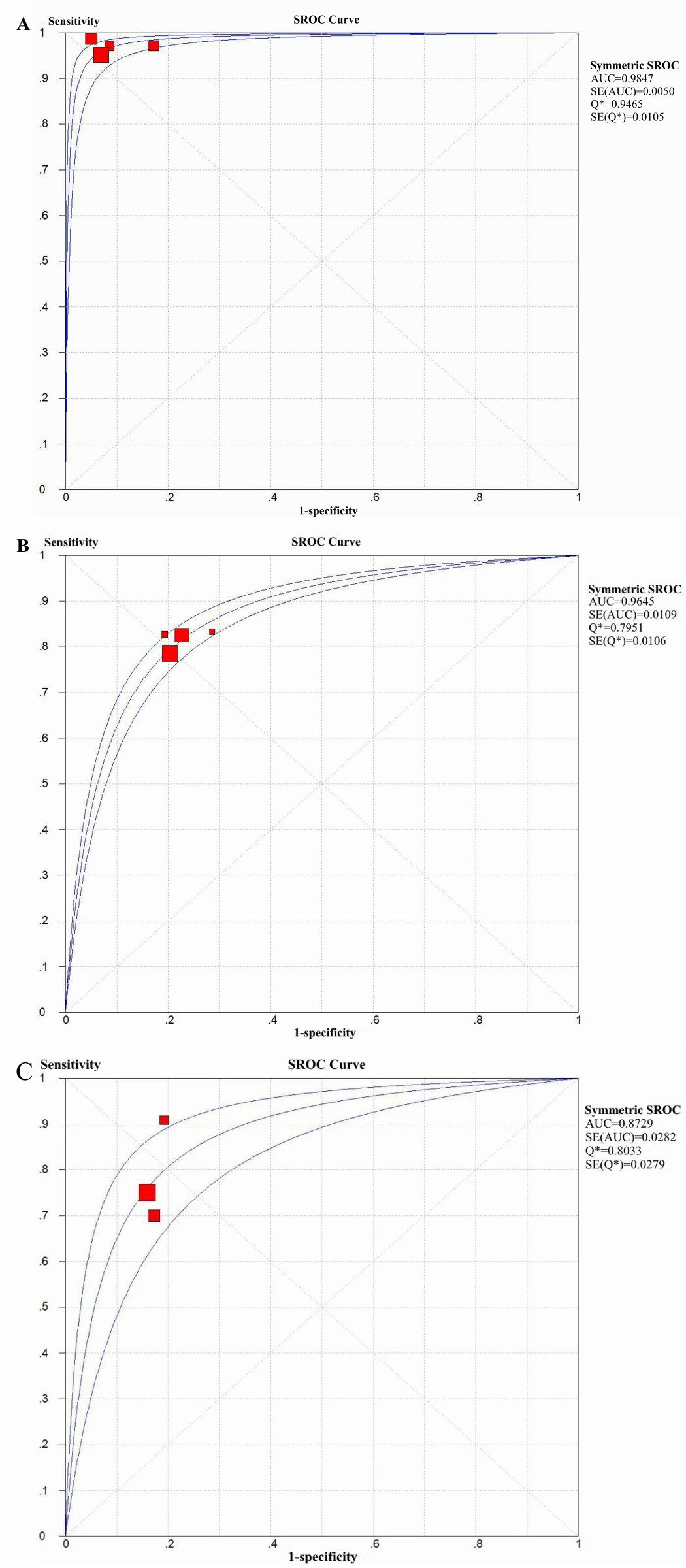


A. MMP-7 for BA diagnosis; B. GGT for BA diagnosis; C. APRi for post-KPE cirrhosis.

All SROC curve are charted via Mantel-Haenszel Fixed Effect Model due to no significance of their weighted regression analysis.
